# Impact of chitosan administration on titanium dioxide nanoparticles induced testicular dysfunction

**DOI:** 10.1038/s41598-022-22044-z

**Published:** 2022-11-16

**Authors:** Amal A. Halawa, Gehad E. Elshopakey, Mohammed A. Elmetwally, Mohamed El-Adl, Samah Lashen, Nancy Shalaby, Ehab Eldomany, Ahmed Farghali, Mohamed Z. Sayed-Ahmed, Nawazish Alam, Nabeel Kashan Syed, Sarfaraz Ahmad, Shaymaa Rezk

**Affiliations:** 1 Department of Forensic Medicine and Toxicology, Faculty of Veterinary Medicine, Mansoura University, Mansoura, 35516 Egypt; 2 Department of Clinical Pathology, Faculty of Veterinary Medicine, Mansoura University, Mansoura, 35516 Egypt; 3 Department of Theriogenology, Faculty of Veterinary Medicine, Mansoura University, Mansoura, 35516 Egypt; 4 Department of Biochemistry and Chemistry of Nutrition, Faculty of Veterinary Medicine, Mansoura University, Mansoura, 35516 Egypt; 5 Department of Cytology and Histology, Faculty of Veterinary Medicine, Mansoura University, Mansoura, 35516 Egypt; 6 Department of Forensic Medicine and Clinical Toxicology, Faculty of Medicine, Damietta University, Damietta, 34517 Egypt; 7 Department of Biotechnology and life sciences, Faculty of Postgraduate Studies for Advanced Sciences (PSAS), Beni-Suef University, Beni-Suef, 62511 Egypt; 8 Material Science and Nanotechnology Department, Faculty of Postgraduate Studies for Advanced Sciences (PSAS), Beni-Suef University, Beni-Suef, 62511 Egypt; 9 Department of Pharmacy practice, college of Pharmacy, Jazan University, Jazan, 82722 Saudi Arabia

**Keywords:** Molecular biology, Physiology, Environmental sciences, Medical research

## Abstract

The potential reproductive toxic effects of oral TiO_2_ NPs in adult male rats as well as the possible alleviation of chitosan administration was investigated. Animals were allocated to four groups; the first group received deionized water and was assigned as a control group. In the second group, rats received chitosan at a dose of 5 mg/kg BW/day. The third group was designed for administration of TiO_2_ NPs at a dose of 150 mg/kg BW/day (1/80 LD_50_). Rats in the fourth group received both TiO_2_ NPs and chitosan. After 14 days, TiO_2_ NPs induced testicular lipid peroxidation as well as oxidative stress. Nano-titanium significantly upregulated genes that encode apoptosis and inflammation in testicular tissue. Moreover, it induced histological alteration in the testicular structure with impairment in spermatogenesis via reduction of PCNA immune-staining. Chitosan administration significantly improved the activities of testicular GPx, SOD, and CAT enzymes. In addition, it significantly down-regulated the relative expressions of pro-apoptotic and pro-inflammatory testicular genes. Chitosan was able to improve the testicular architecture as well as spermatogenesis. The current study revealed the capability of chitosan to ameliorate nano-titanium induced testicular toxicity. Thus, attention should be given to the extensive consumption of nano-titanium particles.

Nanomaterials gain advantages since they cross various body barriers for therapeutic applications as well as their integration into many industrial products, including medical devices, commercial products, and electronics. Nevertheless, this technology has some negative health issues in humans and animals^1,2^. Among nanoparticles, titanium dioxide nanoparticles (TiO_2_ NPs) are widely used in various oral- and dermal consumed products, therefore the exposure to these nanoparticles is unavoidable. Several studies were established for investigation of the toxic impact of TiO_2_ NPs on different body organs, particularly those relating to reproductive issues^3,4^. Reproduction is the way to preserve the species, thus keeping the ecosystem balanced.

Reproductive organs are very sensitive to nanoparticles. It is well documented that TiO_2_ NPs are able to cross the blood–testes and blood–brain barriers^5–7^. TiO_2_ NPs have been validated to accumulate in organs and result in toxicity, with inadequate data regarding male reproductive toxicity caused by TiO_2_ NPs^8^. TiO_2_ NPs can cross the blood-testis barrier to reach the testis and accumulate, resulting in testicular lesions, shifts in serum sex hormone levels, and cell apoptosis^3,4^. Intravenous TiO_2_ NPs induced testicular dysfunction via reduction in testosterone hormone, induction of oxidative stress and apoptosis with bioaccumulation of TiO_2_ NPs in testicular cells of mice^9^.

Chitosan is a natural substance used in medical applications for therapeutic purposes. It is derived from several types of the compound chitin, which is widely found in the outer shells of crustacean species such as shrimp and crabs^10^. Owing to its unique physical and chemical properties, chitosan gained more interest and was integrated into a wide range of applications in the medical field as an antibacterial agent, a drug carrier, and wound healing^11^. Chitosan improves the sperm quality of lead acetate-intoxicated rats^12^.

Reliant on oral LD_50_ of TiO_2_ for rats (> 12,000 mg/kg BW; WHO 1969), we investigated the influence of sub-acute oral TiO_2_ NPs (1/80 LD50) as well as the possible ameliorating effect of chitosan on various testicular biomarkers including oxidative stress/antioxidant parameters, inflammation, and apoptosis in the testes of adult rats.

## Results

### Characterization of TiO_2_ NPs

**Figure 1:**
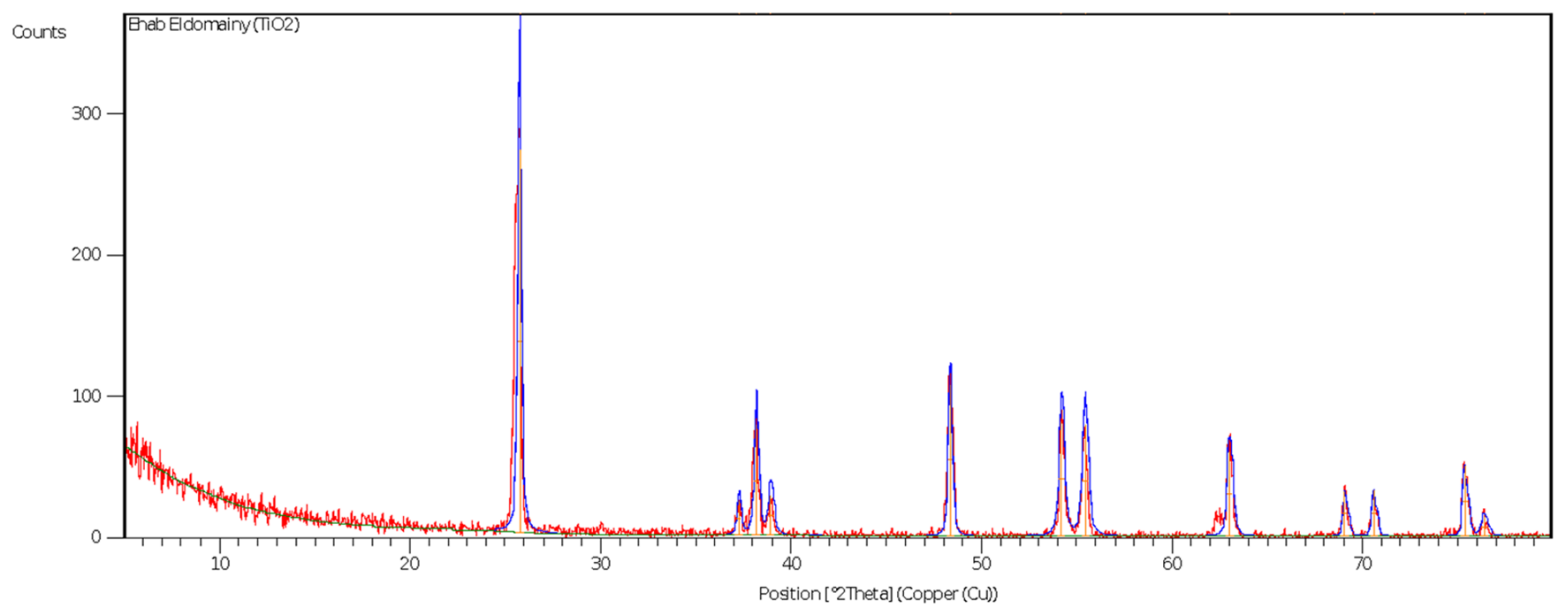
Characterization of TiO_2_ nanoparticles, X-ray diffraction (XRD) peak of crystalline powder of TiO_2_.

**Figure 2:**
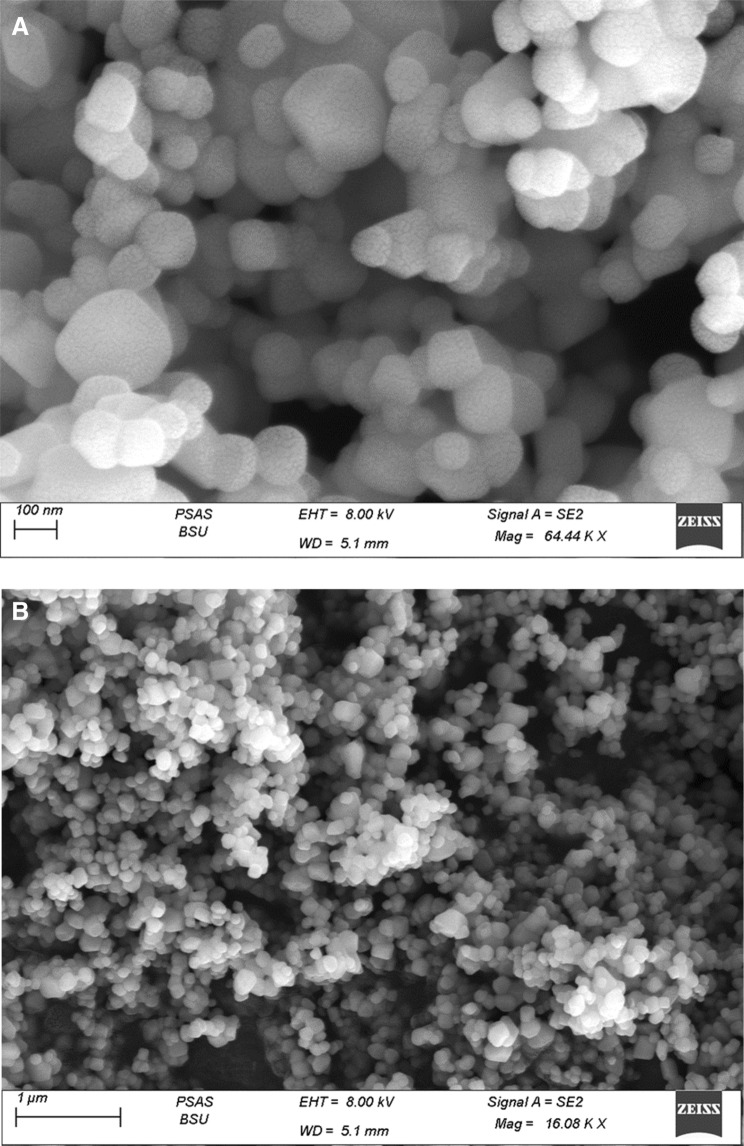
**A**: HRSEM image of a TiO_2_ nanoparticle. **B**: HRSEM image of a TiO2 nanoparticle.

**Figure 3:**
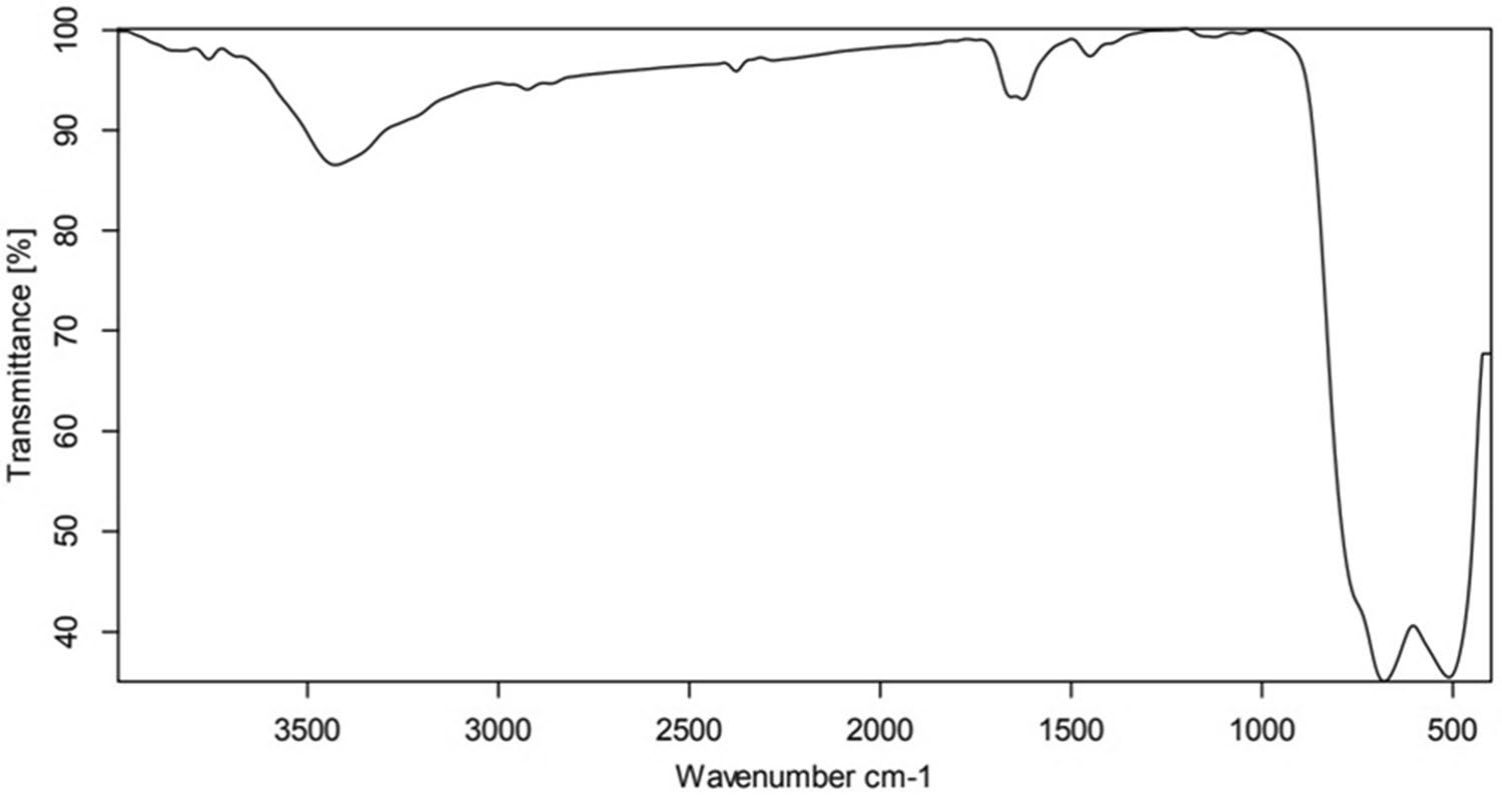
FTIR of TiO_2_ Nanoparticles.

Titanium dioxide (TiO_2_) powders were prepared by ball milling; the crystalline powder of TiO2 was confirmed by X-ray-diffraction (XRD) also, the size of TiO_2_ nanoparticles was average 50–55 nm (Fig. 1). a HRSEM images of a TiO_2_ nanoparticle showed structure, distribution, and size at 100 nm (Fig. 2A) and 1 µm Scale (Fig. 2B). Fourier-transform infrared (FTIR) spectra for Tio2 showed the peaks only corresponding to TiO_2_ at 510 and 680 cm^−1^ (Fig. 3).

### Index weight of testis

Rats that received TiO_2_ NPs showed no alteration in the index weight of their testes (*p* > 0.05) (Table 1).

**Table 1:** Effect of TiO_2_ NPs/Chitosan on testicular index weight.

Group	Control	Chitosan	TiO2 NPs	TiO2 NPs + Ch
Index weight (%)	0.452 ± 0.197	0.465 ± 0.042	0.452 ± 0.031	0.419 ± 0.035

Data are represented as mean ± SEM.

### Serum testosterone level

**Figure 4:**
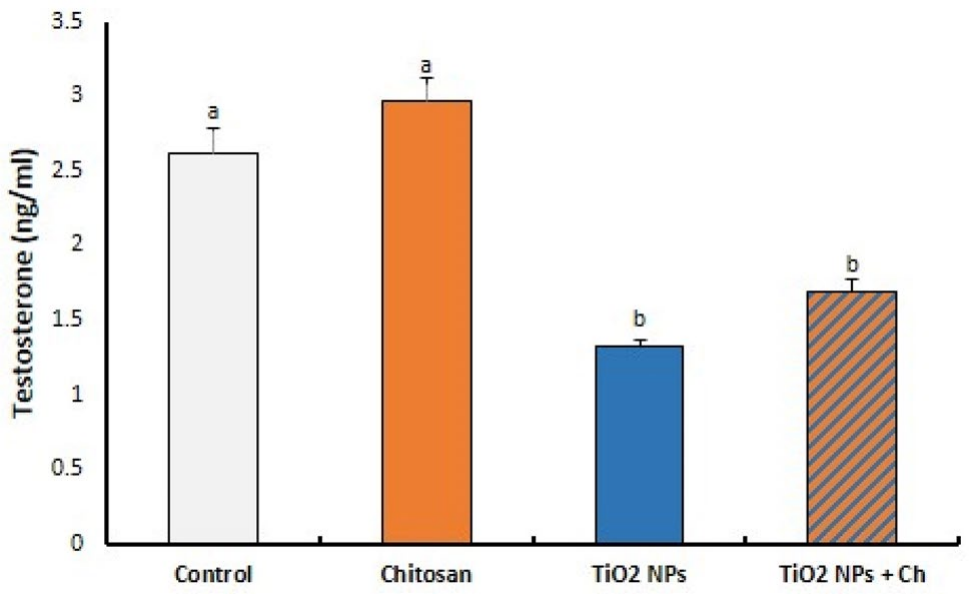
Effects of TiO_2_ NPs and/or chitosan on serum level of testosterone hormone. Data are presented as means ± SEM.

Nano titanium particles significantly reduced serum levels of testosterone hormone compared to the control group (*p* < 0.05). Chitosan showed no obvious effect on testosterone hormone either alone or combined with TiO_2_ NPs (Fig. 4).

### Testicular oxidative/antioxidant biomarkers (Fig. 5)

**Figure 5:**
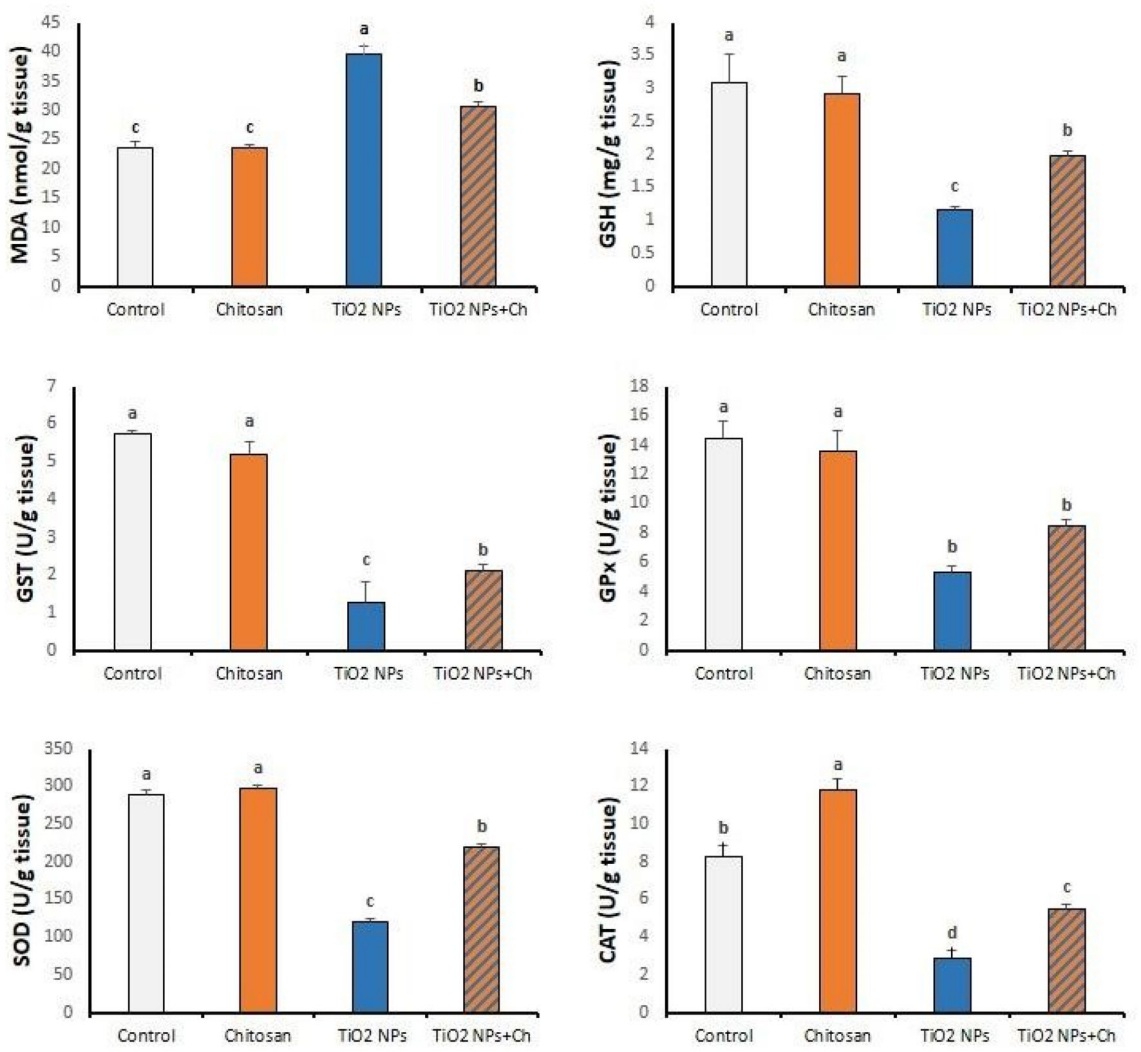
Effects of TiO_2_ NPs and/or chitosan on testicular oxidative/antioxidant markers. data are presented as means ± SEM. data are presented as means ± SEM. Data are expressed as mean ± standard error of the mean (n = 5). Means in the same row with different superscripts are significantly different (*p* < 0.05). MDA (malondialdehyde); GSH (Reduced glutathione); GST (Glutathione S transferase); GPx (Glutathione Peroxidase) SOD (superoxide dismutase) and CAT (catalase).

After 14 days, nano titanium particles significantly increased testicular MDA levels compared to the control group. Chitosan treatment significantly decreased MDA levels compared to the TiO_2_ NPs group (*p* < 0.05).

Glutathione (GSH) levels were markedly reduced in the TiO_2_ NPs group compared to the control group. Chitosan significantly alleviated the TiO_2_ NPs-induced reduction of GSH. The activity of GST enzyme was seriously diminished in TiO_2_ NPs intoxicated rats compared with the control group with a significant improvement in testicular activity of GST in TiO_2_ NPs + Ch group compared to single administration of TiO_2_ NPs. Compared to the control group, the activity of testicular GPx enzyme was markedly reduced in TiO_2_ NPs—intoxicated rats in both TiO_2_ NPs and TiO_2_ NPs + Ch groups without significant variation between them.

Nano-titanium particles severely reduced the activity of testicular SOD enzyme compared to the control group (*p* < 0.05). Chitosan significantly mitigated the TiO_2_ NPs-induced reduction in SOD activity in the TiO_2_ NPs + Ch group. The activity of CAT enzyme was enormously declined in the TiO_2_ NPs group compared to the control one. Chitosan significantly increased the testicular activity of CAT in both, chitosan and TiO_2_ NPs + Ch groups compared to the control and the TiO_2_ NPs groups, respectively (*p* < 0.05).

### Relative expression of pro-apoptotic genes (Fig. 6A)

**Figure 6:**
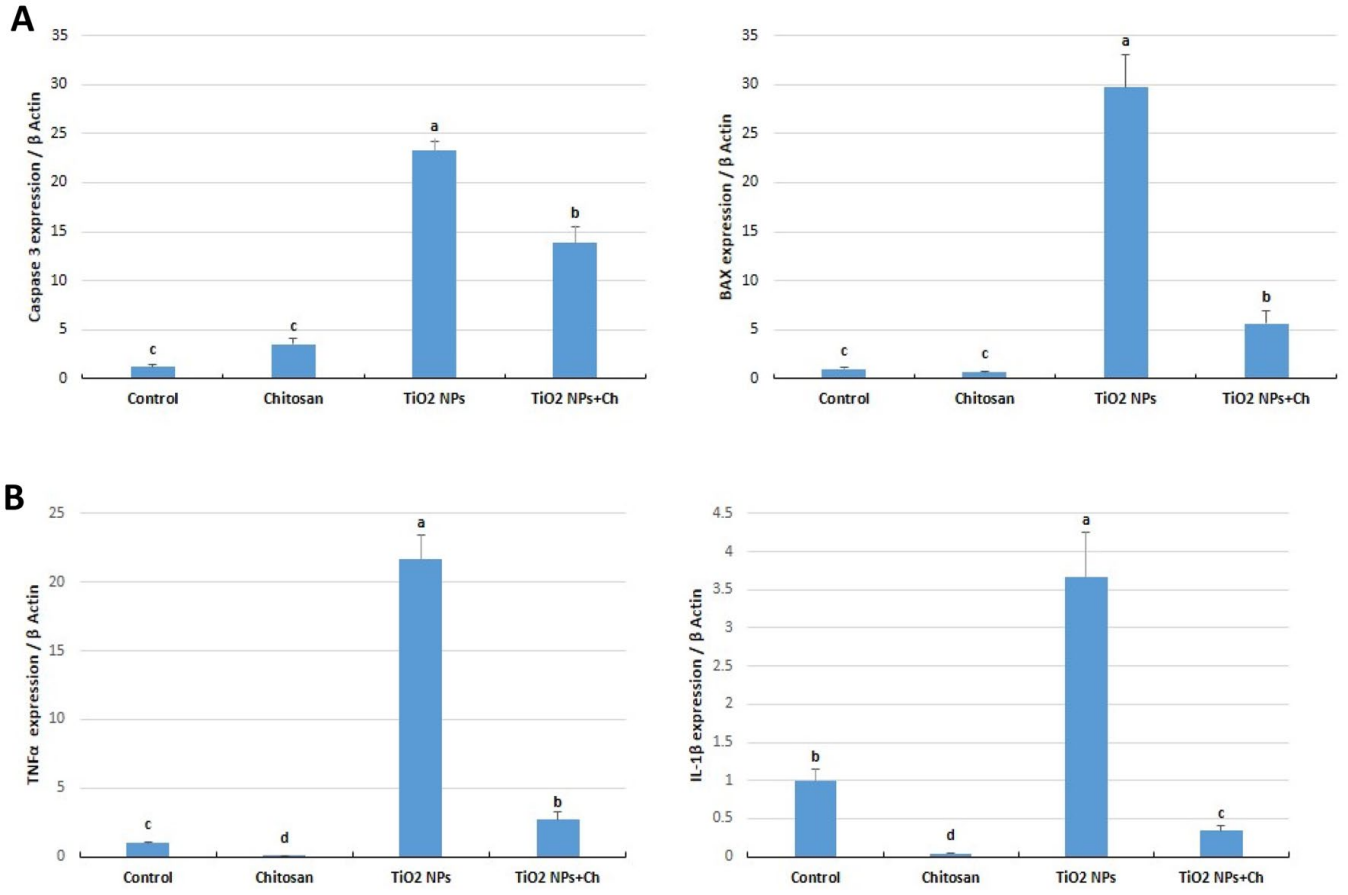
Effects of TiO_2_ NPs and/or chitosan on the expression of pro-apoptotic (**A**: Caspase 3; BAX) and pro-inflammatory (**B**: TNFα and IL-1β) mRNAs. Data are presented as means ± SE. The different letters (a, b, c, d) indicated significant difference (*p* < 0.05) between experimental groups.

The relative expressions of caspase 3 and BAX were immensely up-regulated in the TiO_2_ NPs and TiO_2_ NPs + Ch groups compared to the control group (*p* < 0.05) with a significant alleviating effect of chitosan in the TiO_2_ NPs + Ch group.

### Relative expression of pro-inflammatory genes (Fig. 6B)

Chitosan showed significant downregulation of testicular genes encode inflammation, TNFα and IL-1β, compared to the control group (p < 0.05). Nano-titanium particles extremely upregulated the testicular mRNA expression of both TNFα and IL-1β in the TiO_2_ NPs group compared to the control one (*p* < 0.05). Chitosan treatment significantly downregulated their relative expressions in the TiO_2_ NPs + Ch group compared to single administration of TiO_2_ NPs.

### Histological analysis (H & E stain)

**Figure 7:**
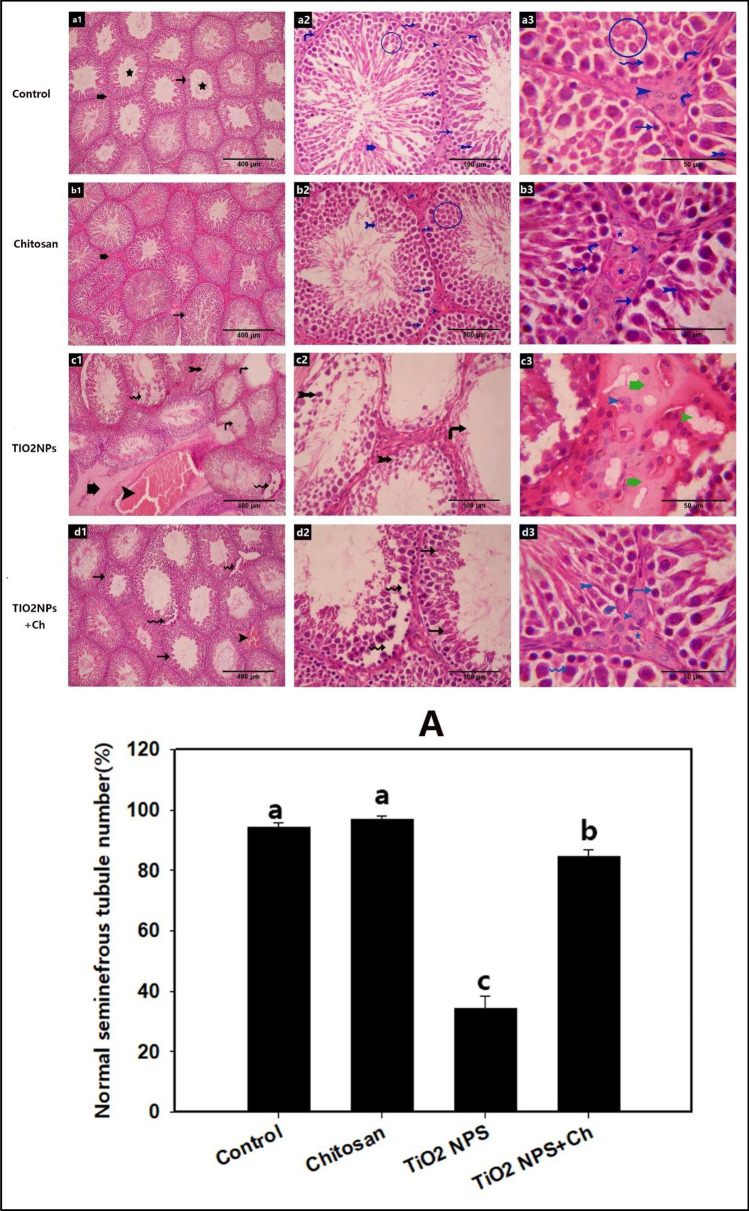
Photomicrograph of a section of rat's testis stained with H&E in different groups: (**a1**–**a3**) control group, (**b1**–**b3**) chitosan group, (**c1**–**c3**) TIO2NPs and (**d1**–**d3**) TIO2NPs+Ch group, showing seminiferous tubules (black thin arrow), interstitial tissue (black thick arrow), lumen of seminiferous tubules(asterisk), myoepithelial cells (blue vertical arrow), spermatogonia (blue thin arrow), primary spermatocytes (blue corrugated arrow), early spermatid (inside blue circle), mature sperm (tailed blue arrow), hyphae of sperm (thick blue arrow), Leydig cells (blue arrow head), Sertoli cells (curved blue arrow), interstitial blood vessels (blue asterisk), seminiferous tubules with disorganized epithelium (black corrugated arrow), shrinkage seminiferous tubules (black vertical arrow), seminiferous tubules with sloughed and necrotic epithelium (black tailed arrow), congested interstitial blood vessels (white arrow). (**A**): % of normal seminiferous tubules, data are expressed as Mean ± SEM. The different letters (a, b, c) indicate significant difference between experimental groups.

As shown in Fig. 5, histological examination of the control group (Fig. 7a1–a3) showed that the testicular parenchyma was studded with numerous normal seminiferous tubules that were separated from the interstitial tissues (Leydig cells and blood capillaries) by a well-defined basal lamina and flat myoid cells. The tubules are lined with 4–8 layers of closely and orderly arranged germinal epithelium (spermatogonia, primary spermatocytes, and spermatids) and Sertoli cells. The histological structure of the testes in the chitosan group (Fig. 7b1–b3) was almost similar to that of the control group (Fig. 7a1–a3). However, in the chitosan group, the seminiferous tubules appeared overcrowded with the germinal epithelium and the interstitial tissues were densely packed with blood capillaries. In the TiO_2_ NPs group, a significant reduction was observed in the number of normal seminiferous tubules with intact germinal epithelium (*p* < 0.05) compared to the control group (Fig. 7c1–c3). Various degrees of germinal epithelium degeneration were detected, ranging from germinal epithelium disorganization, sloughing, detachment, and vacuolization. Moreover, some tubules are irregular, atrophied, and empty of their lining germinal epithelium. Although the interstitial blood vessels were markedly congested and large areas of interstitial tissues appeared vacuolated and covered with pale inflammatory exudates, the nuclei of Leydig cells appeared normal. In the TiO_2_ NPs + Ch group (Fig. 7d1–d3), the mean percentage of preserved seminiferous tubules was significantly increased (*p* < 0.05) compared to the TiO_2_ NPs group (Fig. 7A).

### Spermatogenesis assessment (Fig. 8)

**Figure 8:**
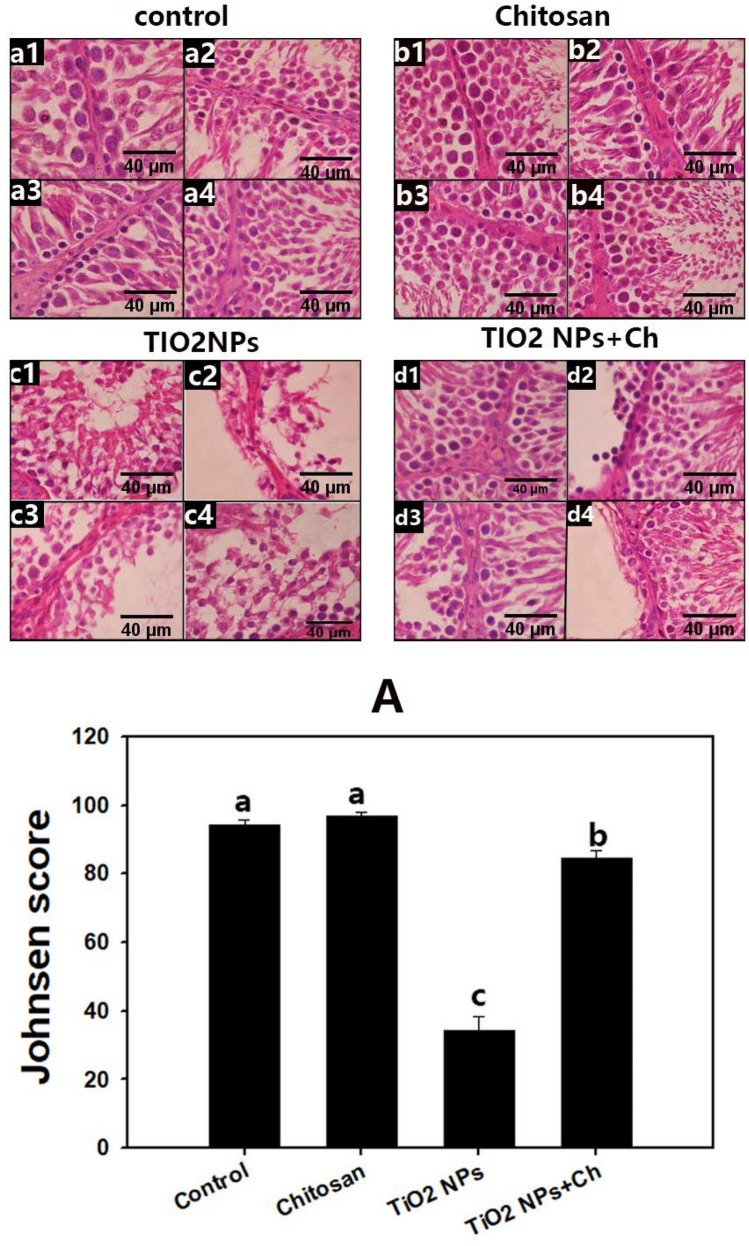
Photomicrograph of a section of rat's testis stained with H&E showing Spermatogenesis assessment of experimental groups (**a1**–**a4**) control group, (**b1**–**b4**) chitosan group, (**c1**–**c4**) TIO_2_ NPs and (**d1**–**d4**) TIO_2_NPs + Ch group. (**A**): Johnson scores of the experimental group, data are expressed as Mean ± SEM. The different letters indicate significant difference (*p* < 0.05) between experimental groups.

Normal spermatogenesis was observed in seminiferous tubules in the control (Fig. 8a1–a4,A) and chitosan (Fig. 8b1–b4,A) groups, without a significant difference in the mean Johnsen's score between them. In the TiO_2_ NPs group, the mean Johnsen’s score was significantly decreased than in the control group, and seminiferous tubules showed maturation arrest (Fig. 8c1–c4,A). In TiO_2_ NPs + Ch group, the majority of tubules achieved a normalized Johnsen's score, with the exception of a small number of tubules with a low score, but the mean score increased significantly in comparison to the TiO_2_ NPs group (Fig. 8d1–d4,A).

### Caspase-3 Immunohistochemical determination (Fig. 9A)

**Figure 9:**
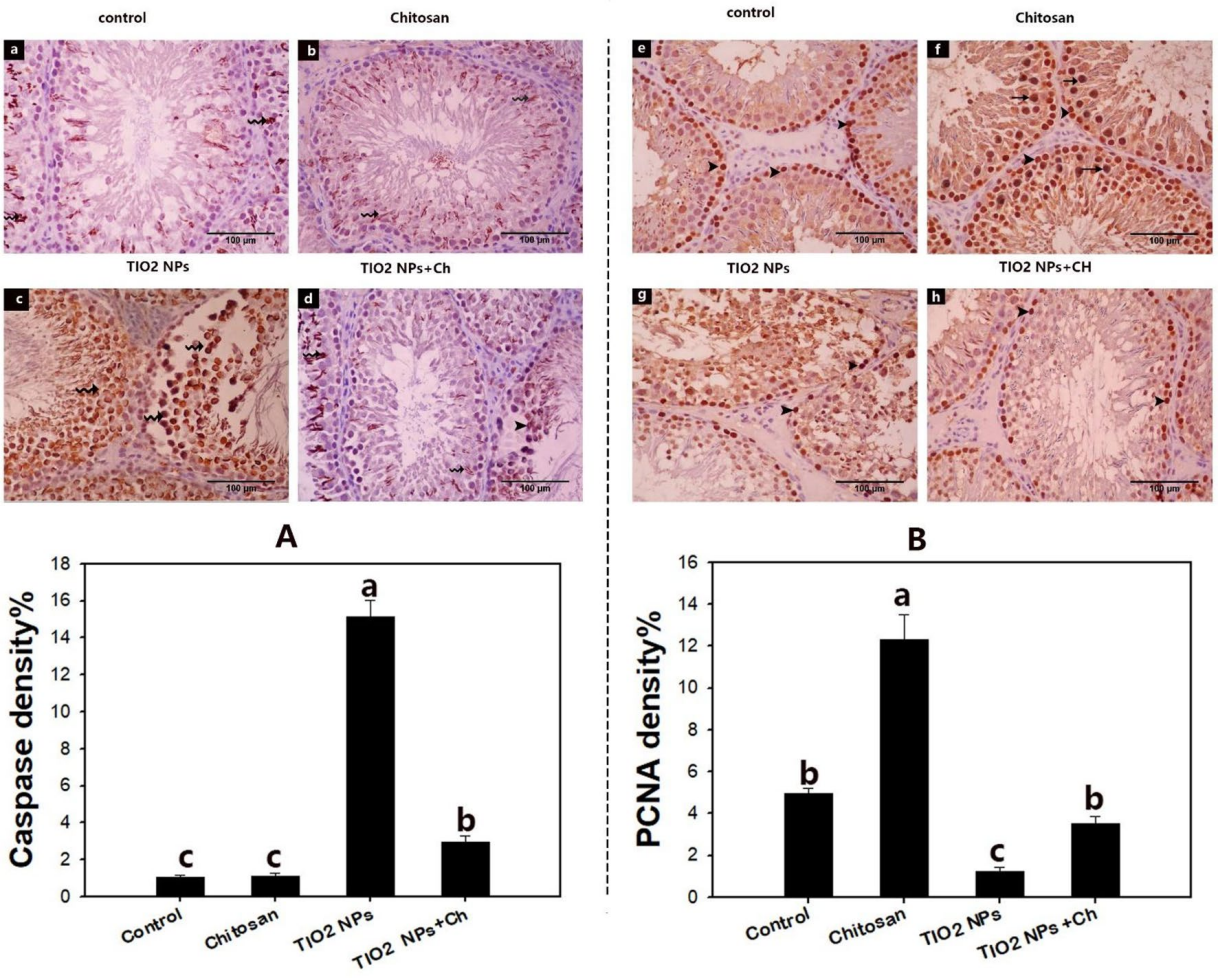
Photomicrograph of Enzyme immunohisto-chemical staining of paraffin sections from a testicular tissue for 1. caspase-3 immune expression (a, b, c and d) showing:only some hyphae of mature sperms in control (**a**) and chitosan (**b**) have positive reaction for caspase (arrow), most of seminiferous epithelium in TIO_2_NPs group (**c**) have positive reaction for caspase (arrow head). In TIO_2_NPs + Chitosan group, some hyphae (arrow) and few spermatocytes (arrow head) have positive reactions for caspase 2. PCNA immune expression (**e**, **f**, **g**, **h**) showing the PCNA expression was detected in the spermatogonia in all groups (arrow head) but in chitosan group was also detected in primary spermatocytes (arrow). (**e**) represent caspase-3 immune expression density, data are expressed as Mean ± SEM. The different letters (a, b, c) indicate significant difference between experimental groups. (**A**, **B**) represent caspase-3 and PCNA immune expression density respectively, data are expressed as Mean ± SEM. The different letters indicate significant difference (*P* < 0.05) between experimental groups.

The density of caspase-3 staining in both the control (Fig. 9a) and chitosan groups (Fig. 9b) showed negligible variation. In the TiO_2_ NPs group, the caspase-3 staining density was significantly increased compared to the control group (Fig. 7c). In the TiO_2_ NPs + Ch group, caspase-3 expression was significantly decreased (Fig. 9d) compared to the TiO_2_ NPs group.

### PCNA Immunohistochemical determination (Fig. 9B)

Expression of PCNA in the control group was quite evident in the nuclei of the spermatogonia (Fig. 9e). Chitosan administration significantly increased the expression of PCNA and was apparently detected in the spermatogonia and primary spermatocytes (Fig. 9f). The expression of PCNA was significantly decreased after TiO_2_ NPs administration (Fig. 9g). In the TiO_2_ NPs + Ch group, the expression of PCNA was significantly increased (Fig. 9h) compared to the TiO_2_ NPs group and approached the PCNA expression in the control group (Fig. 9B).

## Discussion

The aim of the present work was to investigate the potential ability of nano-titanium to induce testicular damage as well as the possible mitigation by chitosan administration.

In the current study, neither nano-titanium nor chitosan affected the index weight of the testes. Likewise, no significance was reported in either body weights or testicular weights in male mice exposed to TiO_2_ NPs at doses up to 100 mg/kg BW for 28 days^13,14^. Mice exposed orally to TiO_2_ NPs at doses of 10, 50 and 250 mg/kg BW from 28 to 70th postnatal day suffered reductions in the body weight gain solely at the highest dose without significant difference in absolute or relative weights of testes^15^. A significant reduction in testicular weight was observed in mice exposed to 300 mg/kg BW of TiO_2_ NPs for 35 days without alteration in body weights^13,14^. Meena et al. 2015^9^ observed a significant decrease in the average coefficients of testis at the higher dose of TiO_2_ NPs (50 mg/kg BW for 30 days), whereas, lower doses of TiO_2_ NPs (5 and 25 mg/kg BW) did not show a significant effect. The reduction in body weight and testicular mass with rising doses of nano-titanium might be attributed to excess accumulation of TiO_2_ NPs in testes^3^. This phenomenon showed that the accumulation of titanium in the organs was closely related to the organ-to-body-weight ratio^9^. Thus, the dose and the exposure time were among the factors that influenced body and organ weights.

After 14 days of exposure time, TiO_2_ NPs significantly reduced serum levels of testosterone hormone without obvious action of chitosan. In consistency, TiO_2_ NPs at doses of 50 or 300 mg/kg BW for 30–35 days significantly decreased serum testosterone levels in rats and mice^9,16,17^. Serum levels of testosterone were significantly reduced after postnatal exposure of mice to 50 and 250 mg/kg/day nano-TiO_2_^15^. Intragastric TiO_2_ NPs for 90 consecutive days passed through the blood–testis barrier and accumulated in the testis, with subsequent testicular lesions, sperm malformations, and alterations in serum sex hormone levels^3^. Testosterone was produced in Leydig cells and in supported Sertoli cells and was required for the attachment of round spermatids to Sertoli cells^18^. Low levels of testosterone might suppress spermatogenesis and cause dysfunction of Sertoli cells^9,19,20^. This reduction may contribute to changes in testis histology and spermatogenesis.

Chitosan nanoparticles at a dose of 280 mg/kg BW for 45 consecutive days attenuated the reduction in testosterone induced by hydroxyapatite nanoparticles^21^. Pretreatment with chitosan nanoparticles (60 mg/kg BW) ameliorated the testicular level of testosterone significantly as compared to the potassium dichromate exposed group^21^. Following 30 successive days, chitosan nanoparticles at a dose of 280 mg/kg in combination with selenium significantly increased serum levels of testosterone in diclofenac–sodium intoxicated rats^22^. In the present work, chitosan might require more exposure time and/or a higher dose to achieve a significant attenuation of the hormonal reduction induced by nano-titanium particles.

In the present study, the testicular level of MDA was obviously increased in the TiO_2_ NPs group, which is compatible with previous studies that were previously established^23^. MDA content serves as an indicator of the extent of lipid peroxidation and is an indirect reflection of the extent of cell damage^14^. MDA is the decomposition product of polyunsaturated fatty acids, and its increase is a result of significant accumulation under high antioxidant stress^14^. Carboxy methylated chitosan significantly reduced testicular lipid peroxidation in the TiO_2_ NPs + Ch group compared to the TiO_2_ NPs group, suggesting the capability of chitosan as a natural biomaterial to alleviate the harm induced by nano titanium particles.

Testicular antioxidant system comprises enzymatic and non-enzymatic constituents. The enzymatic components include SOD, GST and GPx enzymes. The defense mechanism involves conversion of superoxide anion to hydrogen peroxide that is eliminated by CAT or GPx. While GST facilitates conjugation of glutathione with xenobiotic agents for their excretions as a detoxification process^24^. Nano-titanium particles adversely affected the testicular antioxidant system through reductions in GST, GPx, SOD and CAT activities. Similarly, testicular SOD activity was decreased in mice exposed to 100 mg/kg BW TiO_2_ NPs for 28 days^14^**.** Intravenous TiO_2_ NPs for 30 days significantly decreased the SOD and GPx activities at 25 and 50 mg/kg BW with an increase in CAT activity in Wister rats^9^. At a higher dose (300 mg/kg), TiO_2_ NPs enhanced oxidative stress via increasing testicular levels of MDA and reducing testicular levels of SOD, CAT, and GSH^16^. Similar outcomes were reported at the cellular level using rat, human, or prepubertal porcine primary cultured Sertoli cells^25,26^. Nano-titanium particles stimulated production of reactive oxygen species in mouse testes after a 60-day exposure period with a subsequent alteration in the activities of spermatogenesis-dependent enzymes^27^. Thus, oxidative stress and lipid peroxidation might be attributed to the production of free radicals in the testicular tissues and are vital mechanistic paradigms to explain the toxic effects of TiO_2_ NPs.

Chitosan was able to ameliorate testicular oxidative stress induced by TiO_2_ NPs, with a significant increase in CAT activity compared to the control group, confirming its antioxidant properties.

Chitosan increased GSH activity and exhibited antioxidant properties by preventing the decrease in CAT and GSH levels caused by sodium fluoride^28^. In addition, nano-chitosan with hydroxyapatite nanoparticles increased the activities of GPx, GST, CAT, SOD, TAC, and GSH in male rats^21^**.** Chitosan-NPs reversed the diclofenac–sodium mediated decrease in testicular antioxidant markers, SOD and CAT^22^**.** In accordance, Chitosan significantly lowered lead-induced testicular oxidative stress^29^. The pretreatment with nano chitosan (60 mg/kg) significantly decreased MDA levels and increased the activity of GPx levels^23^. Chitosan has a potent antioxidant activity and free radical scavenger potential, which can decrease the lipid peroxidation (MDA level) and increase the antioxidant defense system, which protects against free radical attack with subsequent nullifying of TiO_2_ NPs-induced testicular damage.

Regarding the relationship between antioxidant defense enzymes and apoptotic factors. Glutathione and glutathione-related enzymes are decreased in both their levels and activities after the incidence of apoptosis^30^, as the depletion of GSH is critical for cellular death^31^. Moreover, GST is an enzyme that is required for the protection against DNA damage induced by apoptosis, where exhaustion of GST activity can be found due to the incidence of apoptosis. Superoxide dismutase is an enzyme that is associated with a defense mechanism against free radicals, found to decrease in the case of prostate carcinoma^32^, and found to be effective when supplemented to decrease apoptosis. The downregulation of catalase as well as the decrease in its activity might inhibit the process of apoptosis^33^; due to overproduction of hydrogen peroxide. The overexpression of both catalase and Cu/Zn superoxide dismutase decreased the incidence of apoptosis by 40% due to the decrease in caspase 9^34^. In the current study, the administration of TiO2 can result in the propagation of reactive oxygen species that induce the incidence of apoptosis in testicular tissue as a result of the increase of the degree of lipid peroxidation^35^. Moreover, proinflammatory cytokines such as TNF-alpha can contribute to the induction of apoptosis through stimulation of both c-ABL and p73 pathways during the degradation of retinoblastoma protein^36^.

In the present work, short term oral exposure to TiO_2_ NPs extremely induced testicular inflammation via upregulation of mRNA expressions of TNFα and IL-1β genes with a significant anti-inflammatory action of chitosan. In primary cultured rat Sertoli cells, TiO_2_ NPs induced an inflammatory response via increasing the expression of IL-1β, TNF-α, IFN-γ, and IL-10 in a concentration-dependent manner^37^. Inflammatory mediators present in the normal testis, such as interleukins and TNFα participate in the regulation of spermatogenesis;^38^, therefore the disruption in their expression could perturb normal spermatogenesis. In the current study, TiO_2_ NPs significantly impaired spermatogenesis, evidenced by the reduction that was reported in Johnsen's score.

Our present work showed that the relative expression of both BAX and Caspase 3 was enormously up-regulated in the TiO_2_ NPs exposed group. Likewise, the expression levels of testicular caspase-3, Nrbp2, and cytochrome c and their proteins were significantly increased in mice exposed to oral TiO_2_ NPs (2.5, 5 and 10 mg/kg BW) for 90 days in a dose-dependent manner^39^**.** Significant upregulation of testicular BAX gene expression was detected in rats receiving oral 300 mg/kg BW/day of TiO_2_ NPs for 30 days^16^. Intravenous administration of 50 mg/kg BW/week for 30 days of TiO_2_ NPs significantly activated testicular caspase 3 in exposed rats with a significant reduction in total sperm count suggesting that apoptosis is considered to be involved in the impairment of spermatogenesis and the seminiferous tubules^9^. The pro-apoptotic member of Bcl-2 protein family, BAX, stimulates the mitochondrial pathway, whereas caspase-3 is activated in the apoptotic cell by extrinsic (death ligand) and intrinsic (mitochondrial) pathways^40^. Up-regulation of both BAX and caspase 3 suggests induction of testicular apoptosis via the mitochondrial pathway. A positive correlation was reported between ROS levels and apoptosis in testicular cells. Therefore, oxidative stress encourages apoptotic processes^9,24^. Additionally, the upregulation of the proinflammatory cytokines, IL-1β and TNFα, might have a crucial role in inducing testicular apoptosis via regulating Bcl2 family protein expression^41^.

In the current study, chitosan displayed a testicular anti-apoptotic effect in TiO_2_ NPs + Ch group with respect to the TiO_2_ NPs-intoxicated group. Pretreatment of mice with chitosan nanoparticles significantly decreased potassium dichromate-induced elevation in testicular contents of caspase 3^42^. Oral chitosan nanoparticles at a dose of 600 mg/kg BW for 11 days could protect rat testis from oxidative damage and apoptosis prompted by lead acetate through reducing MDA levels, caspase 3 mRNA expression and, in addition, increasing levels of SOD and GPx^43^.

The present results were confirmed by the histological and morphometric outcomes, including a significant reduction in the number of seminiferous tubules with irregular and atrophied tubules and marked germinal epithelium degeneration. In consistency, in rats exposed to 50 mg/kg TiO_2_ NPs, disorganization and disruption in some seminiferous tubules were observed^9^. Numerous histological alterations were reported in rats exposed to 10 mg/kg of TiO_2_ NPs for 90 days, including irregular arrangement of Sertoli cells in the seminiferous tubules, Sertoli cell apoptosis, necrosis of the seminiferous tubules, decreased thickness of the germinative layer and vacuolation^9^. No histological changes were observed in the testes of mice exposed to 10 mg/kg/day nano-TiO_2_. Whereas, at higher doses (50 and 250 mg/kg/day), seminiferous tubules showed vacuoles with decreased layers of spermatogenic cells in mice that received the high-dose (250 mg/kg/day) of nano-TiO_2_^15^. Nano TiO_2_ particles (50 mg/kg) significant histological alterations in seminiferous tubules, including reduction in their diameter, epithelial vacuolization, sloughing, detachment, and atrophy with abnormal spermatogenesis and significant decline in the Johnsen score^17^. Significant modifications in the testicular morphology of TiO_2_ NP-treated rats might be the result of free reactive radicals and subsequent lipid peroxidation^16^.

Proliferating cell nuclear antigen (PCNA) is a cell cycle regulatory protein marker that is involved in DNA synthesis and has been linked to cell proliferation^44^. PCNA immune-expression in the testis is used as a proliferative marker for spermatogenesis estimation^45,46^. It is considered a rapid, reliable, sensitive, and quantitative approach for determining and detecting early testicular toxicity^47,48^. Spermatogenesis is a dynamic and synchronized process of maturation of stem spermatogonia into mature spermatozoa, which takes place in the seminiferous tubules^49^. In the current study, the testicular PCNA expression was mapped using the immunohistochemistry technique. The significant decrease in the PCNA immune-staining with TiO_2_ NPs highlights the ability of nano-titanium particles to impair spermatogenesis, which is further supported by the histological findings and the significant decline in Johnsen's score. The toxic impact of TiO_2_ NPs on the male spermatogonia has been previously demonstrated^50,51^. The reduction in Johnsen's score in the TiO_2_ NPs group may be attributed to the induction of apoptosis and reduction of the active DNA content in the dividing spermatogonia^52^. The spermatogenic damage in the TiO_2_ NPs group was attributed as well to the induction of lipid peroxidation and oxidative stress that had harmful effects on spermatogenesis^52^.

It is noteworthy that chitosan significantly induced testicular immune-expression of PCNA suggesting its capability to improve spermatogenesis under basal conditions. At the same time, chitosan exerted a potential ameliorative effect counter to that of TiO_2_ NPs through elevations in the regenerated seminiferous tubules, PCNA immune-staining, and a reduction in Caspase-3 expression in respect to the control group. The constructive effect of chitosan on spermatogenesis was obvious as well in raising Johnsen's score compared to the non-treated TiO_2_ NPs group.

This work was the first that combined nano-titanium particles with chitosan to study their effects on various reproductive parameters. Briefly, we should highlight the ability of TiO_2_ NPs to induce testicular dysfunction after short-term of exposure via promotion of inflammation, apoptosis and oxidative stress as well as the reduction in testosterone hormone, the issue that necessitate rising attention for the daily and excessive exposure to oral and occupational nano-titanium particles, and considering its potential toxic impact on reproduction. On the other hand, chitosan was the key to mitigating the adverse effect of TiO_2_ NPs on testicular functions. In the current work, chitosan achieved its role as an antioxidant, anti-inflammatory, and anti-apoptotic biomaterial. In addition, it improved spermatogenesis as well as CAT activity under normal conditions (without TiO_2_ NPs exposure), indicating its ability to upgrade testicular function, an issue that needs further investigation.

## Materials and methods

This study was conducted at Mansoura University with an approved animal use protocol (R/50) in accordance with the Guiding Principles for the Care and Use of Research Animals, Faculty of Veterinary Medicine, Mansoura University, Egypt.

### Chemicals

Titanium dioxide (TiO_2_) was purchased from Sigma-Aldrich Chemical Co., USA. The titanium dioxide nanoparticles, TiO2 NPs, were prepared at the Nanotechnology Unit, Faculty of Postgraduate Studies in Advanced Sciences, Beni-Suef University, according to the methods described by Farghali et al.^46^. The size range of TiO_2_ NPs is less than 60 nm. Solutions of dispersed TiO_2_ NPs were freshly prepared via ultrasonication for 15 min just before oral administration. Carboxymethyl chitosan (CMC, 10%) was purchased from Xin Luk Biotech, China. A field emission scanning electron microscope, FESEM was used to examine the morphologies of the prepared materials (FEI-Quanta FEG-250 SEM). X-ray diffraction, XRD (PANalytical Empyrean, Netherlands) was used to determine phase identification and crystallinity using CuKa radiation (wavelength 1.54045), accelerating voltage of 40 kV, and current of 35 mA. Raman spectroscopy was performed with a Bruker Senterra Raman Microscope (Bruker Optics Inc., Germany). The modified TiO_2_ NPs were successfully disseminated into the chitosan matrix, as determined by analysis using scanning electron microscopy (SEM) and atomic force microscopy (AFM), and the roughness of the chitosan-TiO_2_ nanocomposites was greatly reduced. Additionally, thermogravimetric analysis (TGA) of the thermal characteristics revealed that the chitosan-TiO2 nanocomposites with 0.05 percent TiO_2_ NPs concentration had the best thermal stability. These analyses also revealed that the chitosan interacted with TiO_2_ NPs and displayed good compatibility^53^.

### Characterization of TiO_2_ NPs

Titanium dioxide (TiO_2_) powders were prepared by ball milling; the crystalline powder of TiO2 was confirmed by X-ray-diffraction (XRD) also, the size of TiO_2_ nanoparticles was average 50 -55 nm (Fig. 1). a HRSEM images of a TiO2 nanoparticle showed structure, Distribution and Size at 100 Nm (Fig. 2A) and 1 µm Scale Fig. 2B). Fourier-transform infrared (FTIR) spectra for Tio2 Showed the peaks only corresponding to TiO2 at 510 and 680 cm^−1^ (Fig. 3).

### Animals and treatment

Adult male Albino rats (3–4 months old), purchased from MERC lab (Mansoura University) were kept for acclimatization under standard laboratory conditions (temperature of 22–25 °C, 50–60% relative humidity, and 12 h dark/light cycle) for 7 days. Food and water were available ad libitum.

Animals were randomly distributed into four groups (5 rats each). The first group received deionized water and was assigned as a control group. In the second group, animals received chitosan at a dose of 5 mg/kg BW/day according to Wang et al.^54^. The third group was designed for administration of TiO_2_ NPs at a dose of 150 mg/kg BW/day (1/80 LD_50_) according to Azim et al.^55^. Rats in the fourth group received both TiO_2_ NPs and chitosan (TiO_2_ NPs + Ch) (150 and 5 mg/kg BW/day, respectively). Both TiO_2_ NPs and chitosan were freshly prepared just before administration, following the manufacturers’ instructions. All treatments were given via oral route for 14 consecutive days. On the 15th day, rats were weighed and killed by cervical dislocation. Blood and testes were collected from all groups. Relative testicular weights were calculated using the following equation according to Bearden and Fuquay^56^.

$${\text{Relative}}\;{\text{testicular}}\;{\text{weight}} = \left[ {{\text{testicular}}\;{\text{weight}}\left( g \right)/{\text{Body}}\;{\text{weight}}\left( g \right)^*100} \right].$$

### Serum testosterone level

Serum total testosterone levels were determined following the manufacturer’s instructions of specific kits purchased from Roche-Cobas company (USA; REF. 05200067 190).

### Oxidative/antioxidant parameters

The lipid peroxidation marker, malondialdehyde (MDA), was measured according to Draper and Hadley^57^. Antioxidant defense markers were determined using colorimetric commercial kits [Bio-diagnostic Co, Giza, Egypt]. Reduced glutathione (GSH) concentration was measured colorimetrically using dithionitrobenzoate reagent according to Beutler et al.^58^. Glutathione S transferase (GST) activity was measured according to Habig et al.^59^. The activity of superoxide dismutase (SOD) was measured spectrophotometrically according to Nishikimi et al.^60^.

### Real-time PCR

#### RNA isolation and cDNA synthesis

Testicular tissues were homogenized (100 mg/1 ml) in Trizol™ reagent (Invitrogen, UK) according to manufacturer instructions^61^. The concentration of RNA was detected using a nano spectrophotometer (Quawell, Q5000 UV–Vis spectrophotometer, San Jose, USA). An equivalent of 1 μg of RNA was transferred to cDNA with the High Capacity cDNA Reverse Transcription Kit® (Applied Biosystems) using random hexamers in a 20 µl reaction volume that was further diluted 1:20 for further downstream analysis.

#### Quantification of the immune gene using Real-time PCR

Gene expression was assessed by quantitative RT-PCR. Primers for genes that encode inflammation and apoptosis (http://www.ncbi.nlm.nih.gov/tools/primer-blast/) are listed in Table 2, including their sequences and accession numbers in Genbank.

**Table 2:** Sequences of forward and reverse primers used for qRT-PCR quantitation.

Gene name	Primer sequence	Accession number
Caspase 3	F: GAATGTCAGCTCGCAATGGTAC	NM_012922
	R: AGTAGTCGCCTCTGAAGAAACTAG	
BAX	F: AGACAGGGGCCTTTTTGTTAC	NM_017059.2
	R: GAGGACTCCAGCCACAAAGAT	
TNFα	F: ACTGAACTTCGGGGTGATCG	NM_001278601.1
	R: CCACTTGGTGGTTTGTGAGTG	
IL-1β	F: TGCCACCTTTTGACAGTGATG	NM_008361.4
	R: AAGCTGGATGCTCTCATCAGG	

The application of real-time PCR for amplification and relatively quantifying the specified genes in the current study was conducted on an Applied Biosystem Step One (Thermo Fisher Scientific, UK). Real-time PCR was performed using TOPreal qPCR 2 × premix (enzynomics, South Korea) with the following cycling conditions: Initial denaturation at 95 °C for 8 min, followed by 40 cycles of 95 °C for 40 s, 56 °C for 30 s, and 72 °C for 40 s, then the reaction was terminated by a final elongation cycle at 72 °C for 7 min. The expression analysis was done using the 2ΔΔ ct method adopted by Livak and Schmittgen^62^.

### Histomorphometric and immunohistochemical studies

#### Hematoxylin and eosin staining

Testicular samples were fixed in 10% neutral buffered formalin solution for 24 h. The tissues were then gradually dehydrated with ascending ethanol concentrations, cleaned in xylene, and imbedded in liquid paraffin wax. Using a rotatory microtome, paraffinized blocks were sectioned at a thickness of 5 microns and mounted on either coated glass slides for H & E staining or positive glass slides for immunohistochemical examination^63^.

#### Immune-staining of caspase-3 and proliferating cell nuclear antigen (PCNA)

The technique was applied according to Karen Petrosyan et al. (2002)^64^. Briefly, testicular sections were dewaxed, rehydrated, incubated with 3% hydrogen peroxide at room temperature for 30 min to inhibit endogenous peroxidase activity, and blocked for 15 min with 5% normal goat serum. The sections were then incubated overnight at 4 °C with primary antibodies against either caspase-3 (1:100, 56,046; Santa Cruz Biotechnology, CA, USA) or PCNA (1:500, ab18197; Abcam). Afterwards, the sections were washed, incubated with secondary antibodies, stained with diaminobenzidine, and counterstained with hematoxylin. The mean density of caspase-3 and PCNA expressions was evaluated and expressed as percent using the image analyzer program (version 1.36, NIH, USA).

#### Histomorphometric analyses

Using the light microscope (40 × magnification), morphometric analyses were performed on randomly selected five stained slides for each group (5 fields/slide). The mean percentage of normal seminiferous tubules of the testes^65,66^ was determined using the image analyzer program (version 1.36, NIH, USA). Germinal epithelium maturity was graded according to a modified Johnsen’s scoring method^67,68^. The score, ranging from 1 to 10, was calculated for each animal based on the stage of spermatogenesis. The image analyzer program was used to calculate the mean density of caspase-3 and PCNA immune expression in all groups.

### Statistical analysis

The normality of quantitative parameters (apoptotic and inflammatory gene mRNA expression) was visually examined using normal probability plots and the Kolmogorov–Smirnov test. All data are presented as mean standard deviation of the mean (SEM). Duncan's multiple comparison test was used to perform post hoc multiple pairwise comparisons. The effect of TiO_2_ NPs/chitosan on oxidative stress-antioxidant parameters, expression of apoptotic proteins, and changes in serum testosterone was studied using a mixed model one-way analysis of variance. SAS® was used for statistical analysis (version 9.2, SAS Institute, Cary, NC). For all analyses, the values were considered statistically significant when *p* < 0.05.

### Ethics approval and consent to participate

All used protocols were approved by the Committee on the Ethics of Animal Experiments of the Faculty of Veterinary Medicine, Mansoura University Code No. R/51. All methods were carried out in accordance with relevant guidelines and regulations. The study was carried out in compliance with the ARRIVE guidelines.

## Data Availability

All data generated or analyzed during this study are included in this published article.
