# Author Correction: Impact of chitosan administration on titanium dioxide nanoparticles induced testicular dysfunction

**DOI:** 10.1038/s41598-022-26997-z

**Published:** 2023-01-09

**Authors:** Amal A. Halawa, Gehad E. Elshopakey, Mohammed A. Elmetwally, Mohamed El-Adl, Samah Lashen, Nancy Shalaby, Ehab Eldomany, Ahmed Farghali, Mohamed Z. Sayed-Ahmed, Nawazish Alam, Nabeel Kashan Syed, Sarfaraz Ahmad, Shaymaa Rezk

**Affiliations:** 1 Department of Forensic Medicine and Toxicology, Faculty of Veterinary Medicine, Mansoura University, Mansoura, 35516 Egypt; 2 Department of Clinical Pathology, Faculty of Veterinary Medicine, Mansoura University, Mansoura, 35516 Egypt; 3 Department of Theriogenology, Faculty of Veterinary Medicine, Mansoura University, Mansoura, 35516 Egypt; 4 Department of Biochemistry and Chemistry of Nutrition, Faculty of Veterinary Medicine, Mansoura University, Mansoura, 35516 Egypt; 5 Department of Cytology and Histology, Faculty of Veterinary Medicine, Mansoura University, Mansoura, 35516 Egypt; 6 Department of Forensic Medicine and Clinical Toxicology, Faculty of Medicine, Damietta University, Damietta, 34517 Egypt; 7 Department of Biotechnology and Life Sciences, Faculty of Postgraduate Studies for Advanced Sciences (PSAS), Beni-Suef University, Beni-Suef, 62511 Egypt; 8 Material Science and Nanotechnology Department, Faculty of Postgraduate Studies for Advanced Sciences (PSAS), Beni-Suef University, Beni-Suef, 62511 Egypt; 9 Department of Pharmacy Practice, College of Pharmacy, Jazan University, Jazan, 82722 Saudi Arabia

Correction to:* Scientific Reports* https://doi.org/10.1038/s41598-022-22044-z, published online 16 November 2022

The original version of this Article contained errors in the Affiliations.

Ahmed Farghali and Nabeel Kashan Syed were incorrectly affiliated with Department of Biotechnology and life sciences, Faculty of Postgraduate Studies for Advanced Sciences (PSAS), Beni-Suef University, Beni‑Suef 62511, Egypt.

The correct affiliation for Ahmed Farghali is listed below.

Material Science and Nanotechnology Department, Faculty of Postgraduate Studies for Advanced Sciences (PSAS), Beni-Suef University, 62511 Beni-Suef, Egypt

The correct affiliation for Nabeel Kashan Syed is listed below.

Department of Pharmacy practice, college of Pharmacy, Jazan University, Jazan 82722, Saudi Arabia.

The original version of this Article also omitted an affiliation. The affiliation is listed below:

Material Science and Nanotechnology Department, Faculty of Postgraduate Studies for Advanced Sciences (PSAS), Beni-Suef University, 62511 Beni-Suef, Egypt

As a result, the Affiliations have been renumbered.

The original Article has been corrected.

